# The Nine-Year Survival of Patients Operated for Non-Small-Cell Lung Carcinoma in a Tertiary Centre: The Impact of the Tumour Stage and Other Patient-Related Parameters

**DOI:** 10.3390/medicina60030415

**Published:** 2024-02-28

**Authors:** Silviu Vlăsceanu, Beatrice Mahler, Angela Ștefania Marghescu, Ioana Anca Bădărău, Horațiu Moldovan, Daniela Gheorghiță, Mariana Costache, Cornel Savu

**Affiliations:** 1 Faculty of Medicine, “Carol Davila” University of Medicine and Pharmacy, 050474 Bucharest, Romania; silviu.vlasceanu@drd.umfcd.ro (S.V.); beatrice.mahler@umfcd.ro (B.M.); ancab52@yahoo.com (I.A.B.); mariana.costache@umfcd.ro (M.C.); drsavu25@yahoo.com (C.S.); 2 Department of Thoracic Surgery, “Marius Nasta” Institute of Pneumophysiology, 050159 Bucharest, Romania; 3 Department of Pulmonology, “Marius Nasta” Institute of Pneumophysiology, 050159 Bucharest, Romania; 4 Department of Research, “Marius Nasta” Institute of Pneumophysiology, 050159 Bucharest, Romania; 5 Department of Cardiovascular Surgery, Clinical Emergency Hospital Bucharest, 014461 Bucharest, Romania; 6 Academy of Romanian Scientists, 050045 Bucharest, Romania; 7 Faculty of Material Science and Engineering, National University of Science and Technology Politehnica Bucharest, 313 Splaiul Independentei, 060042 Bucharest, Romania; daniela.gheorghita@upb.ro; 8 Department of Pathology, University Emergency Hospital, 050098 Bucharest, Romania

**Keywords:** lung cancer, surgery, outcome, long-term survival, prognostic factors

## Abstract

*Background and Objectives:* The mainstay treatment of non-small-cell lung carcinoma is still surgery, but its impact on survival beyond nine years has never been reported/analysed in Romania. Therefore, we studied the clinical characteristics and the short- and long-term survival of a population of 1369 patients diagnosed and treated in a single institution, with the variables included in the database being collected retrospectively. *Materials and Methods:* In this paper, we aimed to study a number of factors that might influence prognosis and survival in non-small bronchopulmonary carcinoma. Consequently, we analysed a series of parameters such as the age of patients, their sex, the histopathological type, the tumour stage, the presence of bronchial invasion, and the completeness of surgical resection. *Results:* All patients underwent major lung resection for curative purposes (pneumonectomy, lobectomy, or bilobectomy) between January 2015 and January 2023. The vital status of patients included in the study was obtained by checking the DGEP (General Directorate for Persons Record) database and verifying the reporting of “non-deceased” by the hospital administrative database, as well as by telephone interviews (with patients or their relatives). On univariate analysis, predictors of worse survival were the following: male sex (the hazard of death was 1.54 times higher in men); pT (compared to pT1 tumours, pT2 tumours have a 1.60 times higher hazard of death, pT3 tumours have a 2.16 times higher hazard, and pT4 tumours have a 2.97 times higher hazard); maximum tumour size (a 10 mm increase in tumour size is associated with a 10% increase in the hazard of death); the degree of differentiation (compared to patients with G1 tumours, those with G3 tumours have a 2.16 times higher hazard of death); resectability (compared to R0, R1 B+ has a 1.84 times higher hazard of death, R1 V+ has a 1.82 times higher hazard of death, and R1 B+&V+ has a 2.40 times higher hazard of death). *Conclusions:* As a result, long-term survival can be achieved after complete surgery for NSCLC, and factors that classically predict overall survival suggest that both the initial tumour aggressiveness and host characteristics act beyond the period usually considered in oncology.

## 1. Introduction

Bronchopulmonary cancers are an important cause of morbidity and mortality both in Romania and internationally, being a complex pathology that requires a multidisciplinary approach for early diagnosis and personalized treatment.

At present, the link between smoking and the pathogenesis of this disease is well known, but we should not forget that there is an important category of patients in whom the development of this neoplasia is linked to specific genetic mutations [1,2,3]. Extensive research is currently being performed in this area, all to improve diagnosis and refine therapeutic protocols in order to increase the long-term survival of these patients.

The most common lung cancers are non-small-cell carcinomas (NSCLCs) [1,2], a category in which several histopathological types are included, the most common of which are squamous-cell carcinomas and adenocarcinomas.

The survival of lung cancer patients has been reported in the literature to be 41.8% at 1 year after diagnosis, 12% at 5 years after diagnosis, and only 6.8% at 10 years after diagnosis; the median survival time is 10 months [4], with studies showing that survival is lower in male patients [5].

The prognosis of these patients varies according to different factors: epidemiological, racial/ethnicity, performance status, and the presence of associated conditions, histopathological type and tumour stage (pTNM), the presence of lymphovascular invasion, perineural invasion, spread through air spaces (STAS), and the completeness of surgical resection [1,4].

According to the Global Cancer Observatory (GLOBOCAN) [6], in 2020, Romania reported the death of 54,486 people due to oncological pathologies, with lung cancer being responsible for most deaths (10,779).

## 2. Materials and Methods

The present study is a retrospective, observational, longitudinal, cross-sectional, and non-randomized study, conducted on a sample of 1369 patients diagnosed with curative surgical stage non-small bronchopulmonary carcinoma, who received surgical treatment with curative intent, a representative sample of a population of patients diagnosed with non-small-cell bronchopulmonary neoplasm, treated with curative surgery, diagnosed and treated in a tertiary centre. 

INCLUSION CRITERIA: patients over 18 years of age, diagnosed with primary non-small-cell lung carcinomas in the “Marius Nasta” National Institute of Pneumophthisiology in the period (1 January 2015–1 January 2023), in operable stage with curative intent; patients who have signed the informed consent form at admission.

EXCLUSION CRITERIA: patients diagnosed with other types of primary lung cancers of epithelial and/or non-epithelial origin (small-cell neuroendocrine carcinoma, sarcoma, lymphoma, etc.), lung metastases, carcinomas in situ and benign lung tumours, and non-small-cell lung carcinomas in inoperable stages; patients diagnosed exclusively using biopsy (bronchial, transthoracic, pleural, etc.)/EBUS/cytological methods; patients with neoadjuvant radiation therapy; patients who have not signed/have withdrawn their informed consent; patients operated and/or diagnosed in another medical unit or in a different time frame from the one mentioned above. The main objective of the study was to investigate the role of demographic, and anatomopathological parameters on the evolution and prognosis of these neoplasms. The primary endpoint of the study was OS (overall survival), defined as the period from surgery until the occurrence of death or the date of 1 December 2023. 

Our study was limited by the fact that some of the patients included, who were diagnosed in our Institute with NSCLC based on the examination of the surgery resection specimens, were followed up in the Oncology Departments of other medical care units. Also, some of the patients were lost from our evidence in the context of the SARS-CoV-2 pandemic, due to the difficult access to medical services and/or to the patient’s anxiety about the potential intrahospital infection. This is why we could not focus on the presence of relapses, on the factors that may be associated with relapses, or on relapse-free survival.

For the collection of epidemiological, clinical, and paraclinical information, the internal database of the Institute, the observation sheets of the patients, and the histopathological results from the archive of the Department of Pathology were accessed. We analysed the following variables: the age and sex of the patients, tumour size, the histopathological type of tumour, pTNM stage, lymphovascular, perineural and pleural invasion, the completeness of surgical resection (R), and the survival from the time of diagnosis/operation (overall survival and according to different parameters). 

The collected data were recorded in a database (Excel, version 2401). For the statistical analysis, we used the R software, version 4.2.3 Copyright (C) 2023 The R Foundation for Statistical Computing, R Core Team (2023). R: A language and environment for statistical computing. R Foundation for Statistical Computing, Vienna, Austria. URL https://www.R-project.org (accessed on 5 December 2023).

The following additional packages were used: survival [7], survminer [8], and gtsummary [9]. 

A Kaplan–Meier survival analysis was used, constructing survival curves, the median survival was calculated (both for the overall group and for the different strata determined by the categorical variables in the study), and the survival curves of the different strata were compared using a log-rank test.

A simple Cox regression was also performed, using as predictors the demographic, pathological, and clinical variables followed in the study, with statistically significant predictors then used in a multiple Cox regression.

The α-significance level for the analysis in the study was 0.05, and as such, *p*-values less than 0.05 were considered statistically significant.

## 3. Results

**Figure 1:**
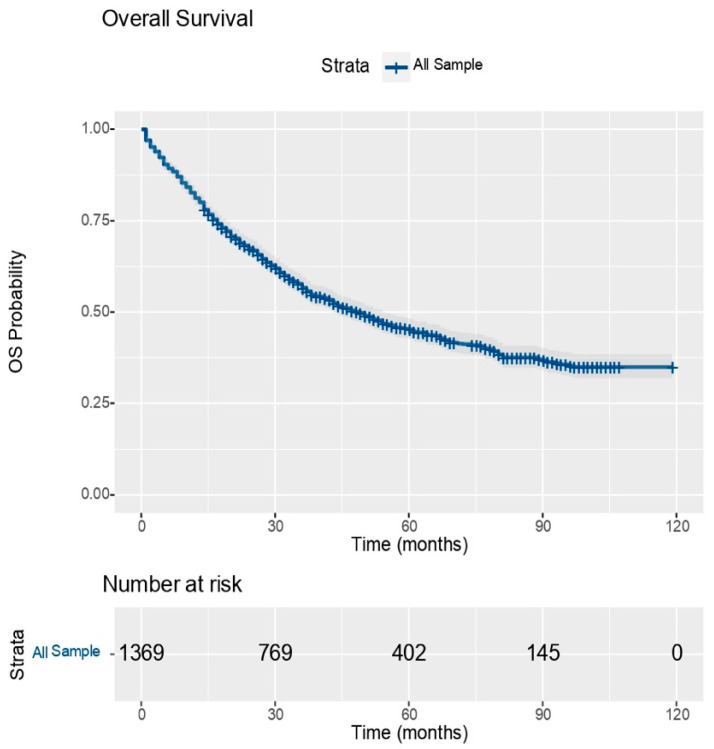
Global OS Graph; source: own contribution.

Figure 1 presents the global OS (overall survival) graph and Table 1 shows the survival analysis on the entire population. Thus, the overall mortality in the group was 55.22%, while the median survival was 49 months.

In Table 2, we analysed a series of parameters (sex, age, pT, pN, maximum tumour size and histopathological type, degree of differentiation, completeness of surgical resection, presence/absence of neoadjuvant treatment, and bronchoscopic appearance) and correlated them with survival.

A positive association was observed between age and the hazard of death, thus a one-year increase in the age of subjects was associated with a 2% increase in the hazard of death. 

From the data obtained, the risk of death was 1.54 times higher in males. Table 3 presents the overall survival of both sexes.

Another parameter that was associated with the survival of NSCLC patients was pT. Compared to pT1 tumours, pT2 tumours had a 1.60 times higher hazard of death, pT3 tumours had a 2.16 times higher hazard, and pT4 tumours had a 2.97 times higher hazard. In determining pT, the maximum tumour size plays an extremely important role, but this is not the only factor involved. A 10 mm increase in tumour size was associated with a 10% increase in the hazard of death.

The degree of tumour differentiation is another factor that was correlated with survival: patients diagnosed with poorly differentiated tumours (G3) had a 2.16 times higher hazard of death than those with well-differentiated tumours (G1).

The completeness of the tumour resection also proved to be very important. Compared to patients with tumours that could be completely resected (R0), the hazard of death was 1.84 times higher in those with a microscopic residual tumour at the bronchial wall (R1 B+), 1.82 times higher in those with a microscopic residual tumour at the vascular resection margin (R1 V+), and 2.40 times higher in those with a microscopic residual tumour at both the bronchial and vascular resection margin (R1 B+&V+).

From Table 4 below, it appears that three-quarters of the patients enrolled in the study were male 75% (N = 1020) and only 25% of the patients (N = 349) were female. 

Evaluating the mortality rate according to sex, we observed that it was higher among men, 59.50% (N = 1020), as opposed to women, 42.69% (N = 349).

**Figure 2:**
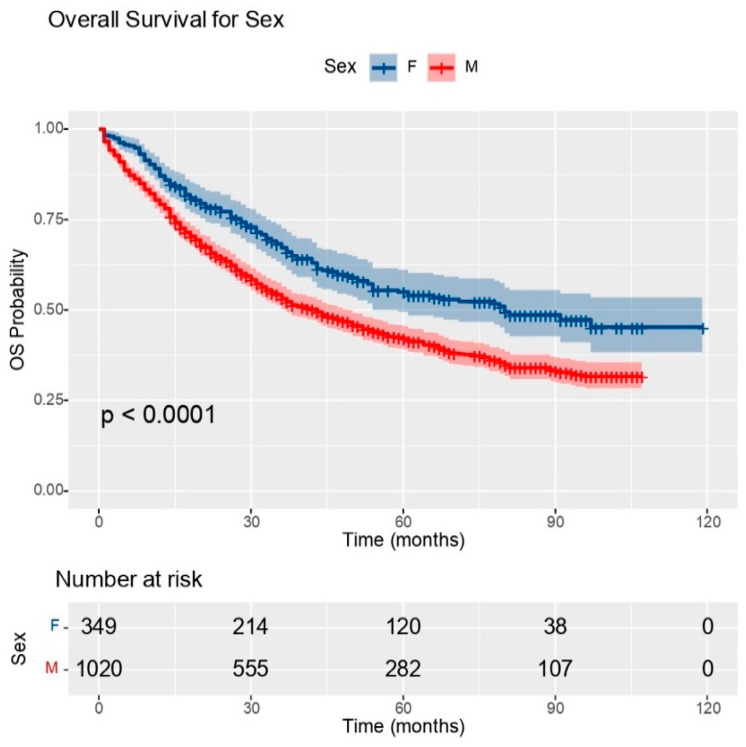
OS graph for the two sexes (OS: overall survival); source: own contribution.

As shown in the graph (Figure 2), there were statistically significant differences in the overall survival of patients according to sex.

The local extension of the tumour is objectified by pT. The more locally advanced the tumour, the higher the mortality rate, an aspect also objectified in our study, where the mortality rate was about one third higher in patients with pT4 tumours compared to those with pT1 tumours. 

The mortality rate was 36.11% for patients with pT1 tumours, 15.09% higher for those with pT2 tumours (51.20%), 23.34% higher for those with pT3 tumours (59.45%), and 33.78% higher for those with pT4 tumours (69.89%) (Table 4).

**Figure 3:**
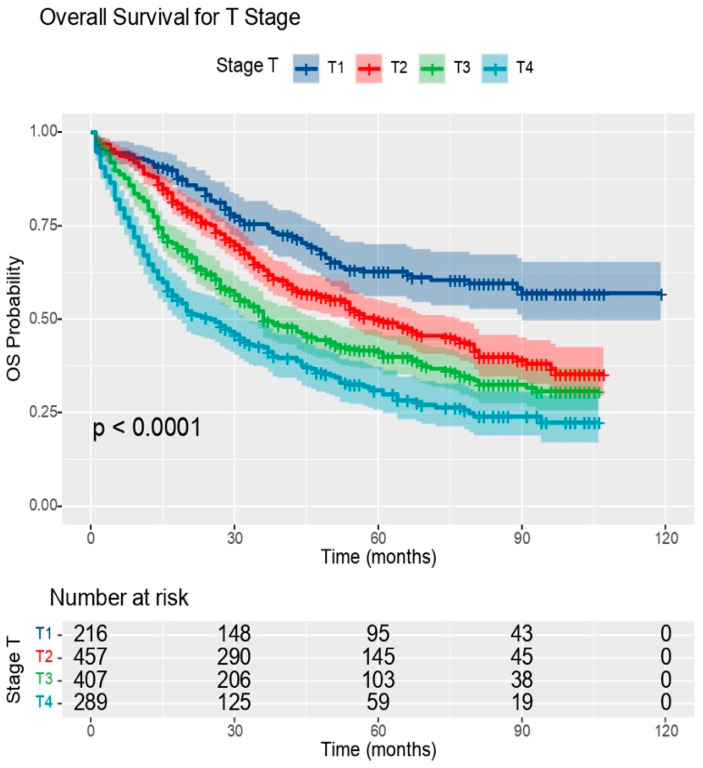
OS graph as a function of T stage (OS: overall survival); source: own contribution.

According to the graph (Figure 3), it is clear that the tumour stage is an extremely important parameter that correlates with the survival of NSCLC patients.

Analysing the data obtained in our study, illustrated in Figure 3, the decrease in survival as a function of pT was observed, including the dynamics over time of these parameters, resulting in the fact that statistically significant differences existed.

**Figure 4:**
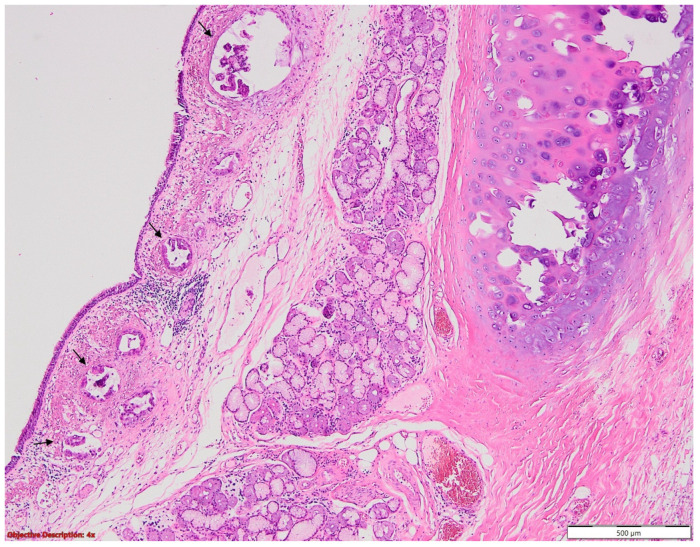
Bronchial resection margin with microscopic tumour infiltration (→) of adenocarcinoma (R1); atypical, neoplastic glands and lymphatic emboli are seen in the lamina propria; HE, 40×.

**Figure 5:**
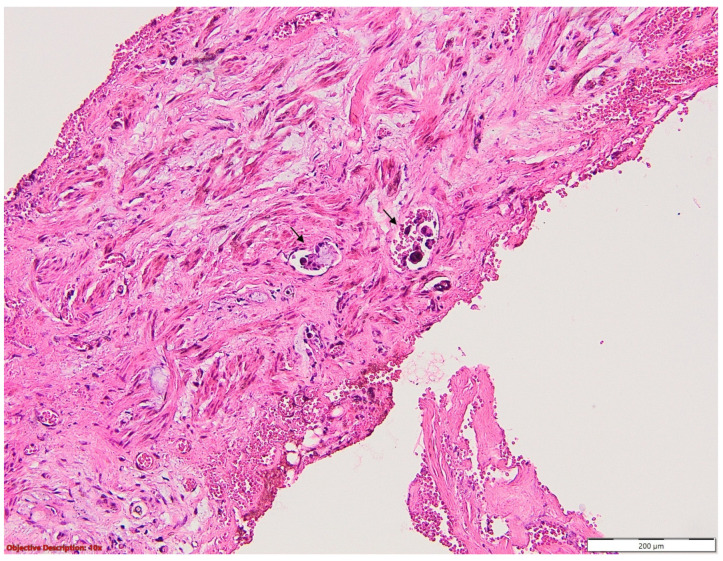
Vascular resection margin with focal microscopic tumour infiltration (R1) (→); rare tumour cells arranged in clusters and micropapillary structures are seen in the lamina propria; HE, 100×.

From Table 5, it appears that of the 1369 patients enrolled, 192 showed residual tumour microscopically, with most (41.66%) having the vascular resection margin infiltrated. However, 39.06% of them showed a bronchial resection margin as presented in Figure 4, that was tumoral, and only 19.28% had both vascular and bronchial margins that were tumour infiltrated. Figure 5 shows vascular resection margin with focal microscopic tumour infiltration.

**Figure 6:**
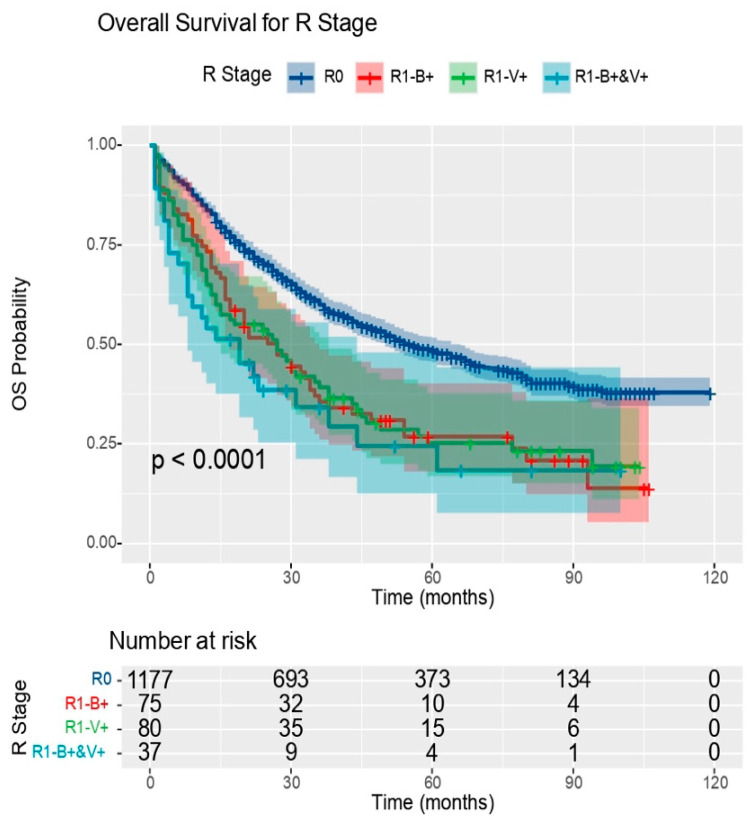
OS Resectability Graph (OS: overall survival); source: own contribution.

Our study aimed at the fact that a residual tumour post surgery influences the survival of patients, the correlation between these parameters being statistically significant. Also, the infiltration of both the bronchial and vascular resection margins had the greatest impact on both long-term and short-term survival (Figure 6).

Analysing the data in Table 6, we outlined a model of five predictors correlated with the survival of subjects included in the study group: age, sex, pT, maximum tumour size, and the completeness of tumour resection.

### Independent Effect of Each Predictor in the Five-Predictor Model

The first predictor of survival was the age of the subjects: there is a positive link between age and hazard of death, and a 1-year increase in age is associated with a 2% increase in hazard of death.

The second predictor was the sex, with the hazard of death being 1.34 times higher in men.

The third predictor of survival was pT. Compared to pT1 tumours, pT2 tumours had a 1.37 times higher hazard of death, pT3 tumours had a 1.6 times higher hazard, and pT4 tumours had a 1.97 times higher hazard. 

The fourth predictor was the maximum tumour size. A 10 mm increase in tumour size was associated with a 5% increase in the hazard of death.

The fifth prognostic factor for survival was the completeness of tumour resection. Compared to patients with tumours that could be completely resected (R0), the hazard of death was 1.89 times higher in those with a microscopic residual tumour at the bronchial wall (R1 B+), 1.65 times higher in those with a microscopic residual tumour at the vascular resection margins (R1 V+), and 2.35 times higher in those with a microscopic residual tumour at both bronchial and vascular resection margins (R1 B+&V+).

In our study, 1369 patients were included (Table 7), three-quarters of whom were male, and the mean age at diagnosis was 62.32 years old. 

Nearly two-thirds of them (63%) were diagnosed with pT2 and pT3 tumours, with the average tumour size in the study group being 5.23 cm.

More than half of the subjects included in the group (57%) had localized disease (pN0). Most of the tumours detected were adenocarcinomas (52%; N = 715), and in terms of differentiation, the majority (52%) were moderately differentiated (G2). We note that data on this parameter were only available for some of the subjects.

Only 6% of patients included in the study population received neoadjuvant therapy. 

In the studied group, 14% of the tumours could not benefit from complete resection; in all these cases, the residual tumour was detected microscopically, most often at the level of vascular resection margins (5.8%).

Analysing the bronchoscopic appearance, the tumour was diagnosed in only about a quarter of cases (26%), not representing a negative prognostic factor.

## 4. Discussion

Bronchopulmonary cancer is the most important cause of death in the population, ranking first among all neoplasms both in incidence and mortality. For this reason, this type of cancer represents a permanent concern in terms of monitoring parameters that would influence survival and prognosis at a distance.

A study conducted by C. Ghiribelli and colleagues correlated the five-year survival rate of a group of 1384 patients diagnosed with NSCLC with the tumour’s stage and the completeness of surgical resection, similar to our study. They noticed that the median survival in the study group was 22 months, without a statistically significant correlation with the histological type of the tumour [10]. When the microscopic residual tumour was objectified, the prognosis was more favourable if the neoplasm was squamous-cell carcinoma compared to adenocarcinoma, similar to other studies (Liewald and colleagues) [11]. The difference, however, was not statistically significant in terms of survival.

In comparison, in our study, conducted over 96 months, the survival rate was 44.88%, with a median survival of 49 months. In terms of the mortality rate according to the histopathological type, same as C. Ghiribelli’s study, we did not observe significant differences, demonstrating that the anatomopathological form would not be a defining parameter for a distant prognosis, as the study group was not large enough to generate feasible results.

Our study sustains (same as Ghiribelli’s research) that the presence of a microscopic residual tumour has a direct impact on distant survival in terms of decreased survival compared to patients (R0) who had a complete surgical resection: the survival rate was 27.09%, compared to 44.8% for the entire population. 

A study by Hofmann and colleagues showed a 14% five-year survival of patients operated for NSCLC, with R1 resection limits; a risk factor analysis showed significant differences according to R1 type (bronchial and extrabronchial) and pN stage [12]. 

Patients with a microscopic residual tumour at the extrabronchial level (including vascular infiltration) had better survival, same as our study. The authors explain this by the fact that R1 represents the consequence of continuous tumour growth in the thoracic wall or vessels of the hilum of the lung. The majority of patients with a microscopic residual tumour at the bronchial level had metastases in the mediastinal lymph nodes, which further worsened the prognosis [12]. 

In our study, most of the R1 tumours (41.66%) presented with vascular invasion (R1 V+), 39.06% had tumour infiltration on the bronchial resection margin (R1 B+), and 19.28% had infiltration both in the vascular and bronchial resection margin (R1 B+&V+). Compared to patients with complete R0 surgical resection, those with residual bronchial tumour infiltration (R1 B+) have a 1.89-fold higher risk of mortality, those with residual vascular infiltration (R1 V+) had a 1.65-fold higher risk, and patients presenting both bronchial and vascular residual infiltration (R1 B+&V+) had the highest risk of 2.35-fold. 

Although techniques for the preoperative assessment of tumour extension have greatly evolved, studies still report a variable percentage, ranging from 1.2 to 17%, of cases in which a neoplastic infiltration of the bronchial resection margin is microscopically detected (WIND J.) [13].

A retrospective study by Dong Lee and colleagues showed that the five-year survival rate was 57.1% and the ten-year survival rate was 40%, correlated with the tumour stage [14]. We obtained a comparable result: the ten-year survival rate in our cohort was 44.88%. 

Other research, such as the study conducted by Riquet and colleagues, revealed a lower survival rate than we found: a five-year survival rate of 44.2% and a ten-year survival rate of 27.7%. 

They also reported that the survival rate of patients depends on the tumour stage and the completeness of surgical resection, including the location of the tumour residue (R1 bronchial/extrabronchial), the same as our study [15].

We observed a 1.54 times higher risk of death in males, the same tendency being noticed by Riquet and colleagues. This would be because, in our cohort, the total number of men was higher, and also because the risk factors are more prevalent among men (e.g., smoking). Therefore, sex would be an indirect prognostic parameter in assessing the survival rate in NSCLC.

In contrast, the tumour stage (pT) has a statistically significant importance; compared to pT1 tumours, pT2 tumours have a 1.60 times higher hazard of death, pT3 tumours have a 2.16 times higher hazard, and pT4 tumours have a 2.97 times higher hazard. A 10 mm increase in tumour size is associated with a 10% increase in the hazard of death. Also, in our study, we observed that the degree of cellular differentiation (G) influences the subsequent evolution of the patient; compared to G1, G3 has a 2.16 times higher risk of death, with the death rate reaching 63.58% of patients.

A study by Kawaguchi and colleagues reported a 14% five-year survival rate for patients with a microscopic residual tumour (R1) at the resection margins [16].

Unfortunately, NSCLC is rarely diagnosed in the early stages, when the five-year survival rate is over 90% after complete tumour resection [17].

A study by Mahdi Sheikh and colleagues found that the five-year survival rate of patients with NSCLC who underwent surgery with curative intent is around 50% in Central and Eastern Europe [18]. This percentage is comparable with the one reported by us.

Similar results have been reported also in research conducted in other geographical areas than ours. A study conducted by Da Som Jeon and colleagues on a South Korean cohort showed a better five-year survival for women with NSCLC, also stage-dependent [19].

Currently, therapeutic options for NSCLC include surgery, chemotherapy, radiotherapy, immunotherapy, targeted therapy, or combinations thereof [20].

TNM staging is a useful tool for predicting the prognosis of patients with NSCLC. Currently, some scientific studies use medical nomograms, and the authors consider them useful for risk stratification in NSCLC patients and for increasing the accuracy of prognostic prediction.

Yi Liu and colleagues mention that there is a need to create a prognostic model based on a thorough assessment of risk factors and survival rates to generate practical guidelines to guide clinicians [21].

The literature highlights the importance of prognostic assessment, especially long-term, in patients with NSCLC, and the need to implement new biomarkers or validated and standardized factors with a prognostic and predictive role. These are extremely important data to guide the treatment plan [22,23].

In contrast to patients diagnosed with NSCLC, those with small-cell lung carcinomas have a lower survival rate of only 2–4 months for untreated tumours and 8–13 months for those who received treatment [24].

A study by Güntuğ Batıhan and colleagues on patients diagnosed with NSCLC found that patient age was an independent variable that influenced the overall survival [25].

In our study, we also found that increased age and tumour stage were independent predictors of long-term survival on multivariate Cox analysis, with a positive association between age and hazard of death showing that a one-year increase in age is associated with a 2% increase in the hazard of death. In addition, adjuvant chemotherapy treatment did not affect the outcome of our study, as the majority of patients in the study (94%) did not receive this type of treatment.

A study conducted by Zijiang Yang and colleagues on 349 patients with stage IB NSCLC showed an overall ten-year survival of 69.6%. The tumour invasion of the visceral pleura was not found to be a useful indicator for prognostic stratification for patients with early diagnosed NSCLC (stage IB), with no statistically significant differences in survival observed in patients with pT1 and pT2a tumours [26].

Other studies have shown that for males, early-stage diagnosis and surgery are protective factors for survival, whilst as far as females are concerned, advanced stages and chemotherapy are risk factors for survival [27]. From our analysis, we could not conclude that chemotherapy treatment would be a risk factor for survival; in our group, adjuvant chemotherapy encompassed only 6% of patients; however, the topic remains open for discussion.

The presence of residual tumour after surgery affects the prognosis [28].

Frozen-section examination guides the surgeon’s medical judgment regarding the bronchial resection margin, but studies report a rate of false-negative results of up to 41.7% due to artefacts generated by processing or due to the possibility of evaluating a small number of sections [29].

There are few studies (such as the one led by C. Lequaglie) that have reported no decrease in long-term survival in patients with NSCLC (stage I, II, and III disease), which is similar in individuals with a microscopic tumour invasion of the resection margin compared to those for whom the tumour has been completely resected [30].

Many of the articles we found evaluated the survival of lung cancer patients at three or five years from the diagnosis moment. The three-year survival rate of patients with incomplete resected NSCLC ranges from 0% to 22% [31].

A study by Tae Hee Hong and others concluded that the extension of microscopic invasion at the bronchial resection margin may be useful for predicting recurrence risk and therapeutic management in patients with NSCLC [32,33].

## 5. Conclusions

Surgical treatment in NSCLC cancer can ensure a good long-term survival rate and can still be considered the main option in this type of pathology. However, this depends on several parameters that directly influence long-term survival such as tumour stage and size, the degree of histopathological differentiation, and the accuracy of surgical resection, which can have effects beyond the standard period usually considered in oncology.

The presence of microscopic residual bronchial, vascular, or associated tumour-tissue negatively influences the prognosis and thus distant survival. Our study revealed interesting information regarding the long-term survival of the patients with incomplete resection. Analysing the type of the microscopical residual tumour, a similar hazard of death was noticed when only one resection limit was involved (regardless of the vascular or bronchial localisation). 

Also, several independent parameters such as age, sex, or histological type may influence long-term survival. Identifying these prognostic parameters helps to identify patients at risk, to consequently use adjuvant therapy. Nevertheless, the study of survival parameters in NSCLC remains an open field and a multidisciplinary approach is the most appropriate.

The particularity of our study lies in the fact that most of the patients (94%) (N = 1285) received only surgical treatment with curative intent, and only a small percentage of 6% (N = 84) of patients had adjuvant chemotherapy treatment associated with the surgery. 

The quality of the study and the prognostic parameters followed are directly influenced by and are closely related to the outcome of long-term surgical treatment.

There are still difficulties regarding traceability in healthcare and the implementation of a National Cancer Register, in which medical professionals could report each case of lung malignancy, following a standard, uniform, and accurate reporting form. In this context, it is difficult to collect extensive epidemiological, clinical, imagistic, and histopathological information, and especially to integrate them with long-term follow-up. We believe that sustained efforts should be made in this direction. 

## Figures and Tables

**Table 1:** Overall survival analysis on the entire population.

Strata	N Deaths (%)	Median Survival (95% CI)
Global	756/1369 (55.22)	49.00 (43.00 to 54.00)

**Table 2:** Simple Cox regression.

Predictor	N	N Deaths	HR (95% CI) ^1^	*p*-Value
Age	1369	756	1.02 (1.01 to 1.03)	<0.001
Sex				
F	349	149	-	
M	1020	607	1.54 (1.29 to 1.84)	<0.001
pT stage				
T1	216	78	-	
T2	457	234	1.60 (1.23 to 2.06)	<0.001
T3	407	242	2.16 (1.67 to 2.79)	<0.001
T4	289	202	2.97 (2.29 to 3.86)	<0.001
pN stage				
N0	780	424	-	
N1	273	152	1.08 (0.90 to 1.31)	0.389
N2	316	180	1.08 (0.91 to 1.29)	0.377
Tumour size	1369	755	1.10 (1.08 to 1.13)	<0.001
Histopathological type				
ADK	715	394	-	
Large-cell carcinoma	137	75	0.96 (0.75 to 1.23)	0.755
Squamous-cell carcinoma	517	287	0.98 (0.84 to 1.14)	0.794
Degree of differentiation				
G1	110	48	-	
G2	335	181	1.28 (0.93 to 1.76)	0.125
G3	195	124	2.16 (1.54 to 3.02)	<0.001
Resectability				
R0	1177	616	-	
R1B+	75	55	1.84 (1.40 to 2.43)	<0.001
R1V+	80	59	1.82 (1.40 to 2.38)	<0.001
R1B+&V+	37	26	2.40 (1.62 to 3.56)	<0.001
Neoadjuvant treatment				
From	84	42	-	
No	1285	714	0.99 (0.73 to 1.35)	0.952
Bronchoscopy				
Negative	1007	548	-	
Positive	362	208	1.07 (0.91 to 1.26)	0.390

^1^ HR = Hazard Ratio, CI = Confidence Interval.

**Table 3:** OS of the two sexes.

Strata Sex	N Deaths (%)	Median Survival (95% CI)
F	149/349 (42.69)	80.00 (54.00 to N/A)
M	607/1020 (59.50)	43.00 (37.00 to 49.00)

**Table 4:** OS by pT tumour stage.

Strata pT Stage	N Deaths (%)	Median Survival (95% CI)
T1	78/216 (36.11)	N/A (90.00 to N/A)
T2	234/457 (51.20)	60.00 (53.00 to 77.00)
T3	242/407 (59.45)	36.00 (33.00 to 47.00)
T4	202/289 (69.89)	25.00 (18.00 to 33.00)

**Table 5:** OS by resectability.

Strata Resectability	N Deaths (%)	Median Survival (95% CI)
R0	616/1177 (52.33)	N/A (90.00 to N/A)
R1-B+	55/75 (73.33)	27.00 (17.00 to 36.00)
R1-V+	59/80 (73.75)	27.00 (15.00 to 38.00)
R1-B+&V+	26/37 (70.27)	19.00 (8.00 to 44.00)

**Table 6:** Multiple Cox regression (all predictors in the model with statistical significance).

Predictor	N	N Deaths	HR (95% CI) ^1^	*p*-Value
Age	1369	756	1.02 (1.01 to 1.03)	<0.001
Sex				
F	349	149	-	
M	1020	607	1.34 (1.12 to 1.61)	0.002
pT stage				
T1	216	78	-	
T2	457	234	1.37 (1.06 to 1.78)	0.018
T3	407	242	1.60 (1.21 to 2.11)	0.001
T4	289	202	1.97 (1.40 to 2.75)	<0.001
Tumour size	1369	756	1.05 (1.01 to 1.09)	0.007
Resectability				
R0	1177	616	-	
R1-B+	75	55	1.89 (1.43 to 2.50)	<0.001
R1-V+	80	59	1.65 (1.26 to 2.16)	<0.001
R1-B+&V+	37	26	2.35 (1.58 to 3.49)	<0.001

^1^ HR = Hazard Ratio, CI = Confidence Interval.

**Table 7:** Descriptive statistics of the variables followed in the study; for continuous variables, the mean and standard deviation (SD) were determined (considering the sample size), whilst for categorical variables, the absolute and relative frequencies were determined.

Variable	N = 1369
Sex, n (%)	
F	349 (25)
M	1020 (75)
Age, Medium (SD)	62.32 (8.38)
pT stage, n (%)	
T1	216 (16)
T2	457 (33)
T3	407 (30)
T4	289 (21)
pN stage, n (%)	
N0	780 (57)
N1	273 (20)
N2	316 (23)
Tumour size, Medium (SD)	5.23 (2.54)
N/A	2
HP type, n (%)	
ADK	715 (52)
Large-cell carcinoma	137 (10)
Squamous-cell carcinoma	517 (38)
Degree of differentiation, n (%)	
G1	110 (17)
G2	335 (52)
G3	195 (31)
Unknown	729
Resectability, n (%)	
R0	1177 (86)
R1-B+	75 (5.5)
R1-V+	80 (5.8)
R1-B+&V+	37 (2.7)
Neoadjuvant treatment, n (%)	
From	84 (6)
No	1285 (94)
Bronchoscopy result, n (%)	
Negative	1007 (74)
Positive	362 (26)

## Data Availability

The data presented in this study are available on reasonable request from the corresponding author.
